# The Ability to Digest Cellulose Can Significantly Improve the Growth and Development of Silkworms

**DOI:** 10.3390/insects15120997

**Published:** 2024-12-16

**Authors:** Jinxin Wu, Yungui Zhang, Han Chen, Qingyou Xia, Ping Zhao, Ying Lin, Guanwang Shen

**Affiliations:** Integrative Science Center of Germplasm Creation in Western China (Chongqing) Science City, Biological Science Research Center, Southwest University, Chongqing 400715, China

**Keywords:** cellulose, transgenic, silkworm, growth, development

## Abstract

Cellulose is essential in the growth and development of herbivores. However, its limited utilization by herbivores is a key factor restricting their feed conversion rates. Cellulase can hydrolyze cellulose into glucose, and the addition of exogenous cellulase preparations to feed is an effective method for improving the cellulose utilization rate of ruminants. Nevertheless, the decomposition efficiency of exogenous cellulase is unstable and susceptible to various external factors. In this study, the endoglucanase III gene from *Apriona germari* (*AgEGase III*) was introduced into silkworms to investigate whether transgenic silkworms with cellulose-digesting capabilities outperformed normal silkworms in terms of growth, reproduction, and economic traits. The results show that the transgenic silkworms exhibited increased body size, weight, feeding efficiency, and digestibility compared to the wild-type silkworms. The cocoon weight, shell weight, cocoon–shell ratio, and pupa weight were notably elevated by 11%, 37%, 23%, and 9%, respectively. Additionally, the egg weight and egg-laying quantity of the female moth were also significantly increased compared to those of the wild type. Furthermore, feeding transgenic silkworms with an artificial feed containing additional cellulose demonstrated their ability to digest and utilize cellulose, leading to improved growth and development. This study offers theoretical support for the development of transgenic ruminant species that express cellulolytic enzymes.

## Simple Summary

Enhancing the ability of herbivores to digest cellulose is an effective method of improving feed conversion rates and increasing animal productivity. In this study, we introduced the endoglucanase II gene from *Apriona germari* into silkworms to determine whether an additional cellulose digestion capability activity changed the growth, reproductive capacity, and economic traits of the now-transgenic silkworms. The results showed that the transgenic silkworms exhibited increased body size, weight, feeding efficiency, and digestibility compared to the wild-type silkworms. Additionally, the economic traits of the transgenic silkworms, the egg weight, and the egg-laying quantity of the female moth were also significantly increased compared to those of the wild-type silkworms. Furthermore, feeding transgenic silkworms with an artificial feed containing additional cellulose demonstrated their ability to digest and utilize cellulose, leading to improved growth and development. This study offers theoretical support for the development of transgenic ruminant species that express cellulolytic enzymes.

## 1. Introduction

Cellulose in coarse feed plays a crucial role in the dietary structure of cattle and sheep, as it is indispensable in the development of ruminants. Cellulose stimulates chewing and salivary secretion, promotes rumination, and enhances intestinal movement and health. Moreover, it aids in buffering rumen acidosis, regulates feed intake, and is crucial in the digestion of solid feed particles in the rumen [1]. Cattle and sheep lack cellulase genes and depend on cellulose-degrading bacteria in the rumen for digestion [2,3,4]. Incomplete cellulose digestion can restrict the breakdown of coarse feed and energy uptake in cattle and sheep, thereby lowering their productivity [1]. Improving cellulose digestion in cattle and sheep is therefore essential. Researchers have improved the utilization of cellulose in cattle and sheep feed by adding exogenous cellulase enzymes [3,5,6,7]. However, the enzymatic efficiency of these exogenous cellulase preparations may be influenced by various factors, such as dosage, enzyme activity, metal ion cofactors, pH, and temperature [8,9,10].

Transgenic technology has played a significant role in animal breeding and genetics. In the 1980s, Hammer et al. (1985) conducted the first genetic modifications in farm animals [11]. Wu et al. (2015) inserted the mouse gene SP110 into the genome of cows to boost their resistance to tuberculosis [12]. Researchers have successfully used transgenic techniques to produce human proinsulin and lactoferrin from the milk of cows, thereby enhancing its nutritional and practical value [13,14]. It may be possible that the introduction of cellulase genes into the genomes of cattle and sheep enhances cellulose digestion and improves feed efficiency. Nevertheless, the application of transgenic technology in farm animals still faces significant limitations due to the complexity of genetic modification techniques in large animals such as cattle and sheep, as well as the ethical and legal considerations involved [15].

Silkworms are important economic insects due to their high level of domestication, short lifecycle, high reproductive rate, ease of rearing, and simple genetic manipulation. They also serve as an ideal model organism for research in the life sciences [16]. Transgenic technology has broad applications in the development of silkworm bioreactors and genetic breeding. Researchers have utilized the silk glands of silkworms as bioreactors to produce recombinant human lactoferrin and bioactive human platelet-derived growth factor, thereby increasing the economic value and potential applications of silkworms [17,18]. Mi et al. (2023) successfully expressed spider silk fibers in domestic silkworms using gene editing technology to produce transgenic silkworms with tough and strong silk [19].

Mulberry leaves, the primary food source for silkworms, contain approximately 13% cellulose [20]. However, cellulose digestion requires cellulase to break it down into cellobiose and glucose [21]. β-Endoglucanase randomly cleaves the β-1,4-glycosidic bonds in the amorphous regions of cellulose, producing smaller cellulose oligosaccharides. Subsequently, β-exoglucanase acts on the terminal ends of these oligosaccharides to yield cellobiose or glucose. Finally, β-glucosidase hydrolyzes cellobiose into two glucoses, thereby completing the degradation of cellulose [22,23]. Silkworms lack β-endoglucanase and β-exoglucanase and therefore cannot digest the cellulose in mulberry leaves [24,25,26]. Fortunately, the mulberry longicorn beetle *Apriona germari*, an insect that feeds on mulberry bark, possesses genes encoding cellulase enzymes, providing it with a robust cellulose-digesting ability [27,28,29].

In this study, we introduced the *AgEGase III* gene from *A. germari* into silkworms. Assuming that AgEGase III remains active when expressed in a different species, this study investigated whether transgenic silkworms with cellulose-digesting capabilities outperformed normal silkworms in terms of growth, reproduction, and economic traits. This study provides theoretical support for the development of transgenic ruminants capable of producing cellulolytic enzymes.

## 2. Materials and Methods

### 2.1. Experimental Insects

The silkworm strain D9L and the artificial feed used in this study were preserved and provided by the Biological Science Research Center at Southwest University, China. The silkworms were reared in an environment with a temperature of 25 ± 1 °C, a 12 h light/12 h dark cycle, and a humidity of 75%. The composition of the artificial feed aligned with that used in a previous study [30].

### 2.2. The Creation of Transgenic Silkworms

The construction of the transgenic vector piggyBac [3 × p3-DsRed-SV40, HR3-A4-AgEGaseIII-SV40] involved ligating the synthetic *AgEGaseIII* gene (GenBank: AY771358.1; Tsingke Biotech, Beijing, China) sequence with the double-digested (*Bam*HI and *Not*I) psl1180-HR3-A4-SV40 backbone fragment to obtain the intermediate vector psl1180-HR3-A4-AgEGaseIII-SV40. Subsequently, primers were designed for the PCR amplification of the HR3-A4-AgEGaseIII-SV40 fragment from the psl1180-HR3-A4-AgEGaseIII-SV40 vector. This fragment was then inserted into the transgenic vector piggyBac [3 × p3-DsRed-SV40] via homologous recombination to create a recombinant transgenic vector. The HR3 is a homologous region from Bombyx mori nucleopolyhedrovirus with a promoter transcriptional enhancement function [31]. The A4 promoter, known for its robust transcriptional activity and frequent utilization in the laboratory [32], controls the expression of *AgEGase III* in silkworms. Furthermore, the red fluorescent protein driven by the eye-specific promoter 3 × p3 served as a screening marker for the identification of transgenic-positive silkworms. The PCR amplification procedure included an initial denaturation at 98 °C for 3 min, followed by 35 cycles of denaturation at 98 °C for 10 s, annealing at 56 °C for 20 s, extension at 72 °C for 40 s, and a final extension at 72 °C for 5 min. PrimeSTAR^®^ Max DNA Polymerase (TAKARA Biotech, Kyoto, Japan) was used for PCR amplification, following the manufacturer’s instructions, while the homologous recombination process was facilitated by the Seamless Cloning Kit (Abm, Shanghai, China). The primers used for PCR were as follows: F, 5′-TTATCGATACGCGTACGGCGCAGCGTCGTGAAAAGAGGCAATGAC-3′ and R, 5′-GAGATCGGCCGGCCTAGGCGTTCGTCAATGTATCAGTTTTGGT-GC-3′.

A mixture of transgenic recombinant plasmid and helper plasmid (with a concentration of 400 ng/µL) was injected into silkworm eggs within 2 h after laying. After injection, the eggs were incubated at 25 °C and fed fresh mulberry leaves. Subsequently, the silkworm eggs and moths were examined under a fluorescence microscope to determine the expression of DsRed-positive signals. Transgenic silkworms were selected and named as AgEGaseIII^OE^ for further experiments.

### 2.3. RNA Extraction and cDNA Synthesis

Midguts from different developmental stages of both wild-type D9L and transgenic silkworms were ground in liquid nitrogen, and the total RNAs were extracted using a TRIzol extraction kit (Invitrogen, Waltham, MA, USA). The extracted total RNA was treated with DNase I (TaKaRa Biotech, Kyoto, Japan) to prevent genomic DNA contamination. First-strand cDNA of the midgut was synthesized using the M-MLV Reverse Transcriptase (Promega, Madison, WI, USA) following the manufacturer’s instructions and stored at −20 °C.

### 2.4. Real-Time PCR (RT-PCR) and Quantitative Real-Time PCR (qRT-PCR)

For the detection of *AgEGase III* in the transgenic silkworm, RT-PCR was performed under the following conditions: 98 °C for 3 min; 30 cycles at 98 °C for 10 s, 56 °C for 20 s, and 72 °C for 15 s; and 72 °C for 5 min. The reaction conditions for *BmActin3* (control) were as follows: 98 °C for 3 min; 30 cycles at 98 °C for 10 s, 56 °C for 20 s, and 72 °C for 10 s; and 72 °C for 5 min. The PCR products were assessed using agarose gel electrophoresis.

Quantitative (q)RT-PCR was used to detect the expression levels of *AgEGase III*. The SYBR Premix Ex TaqTM (TaKaRa Biotech, Kyoto, Japan) and an ABI StepOne v2.1 Sequence Detection System (Applied Biosystems, Waltham, MA, USA) were used. The internal control used was the silkworm translation initiation factor 4A (*BmTIF4A*, NM_001043911.1), and the relative expression level of the target gene was determined using the 2^−∆∆Ct^ method. The PCR amplification program included an initial denaturation step at 95 °C for 5 min, followed by 35 cycles of denaturation at 95 °C for 3 s and extension at 60 °C for 30 s. The specific primers used for the RT-PCR and qRT-PCR were as follows: *AgEGaseIII*, F 5′-GATGCCGAATTGAAGACGA-3′ and R 5′-TCACAGCTTCATGGTACGGT-3′; *BmActin3*, F 5′-AACACCCCGTCCTGCTCACTG-3′ and R 5′-GGGCGAGACGTGTGATTTCCT-3′; and *BmTIF4A*, F 5′-TTCGTACTGGCTCTTCTCGT-3′ and R 5′-CAAAGTTGTAGCAATTCCCTA-3′.

### 2.5. Activity Detection for AgEGase III

The midguts of third-day fifth-instar silkworms were ground thoroughly in liquid nitrogen, followed by the addition of phosphate-buffered saline (PBS), and were thoroughly mixed. A specific amount of the protease inhibitor phenylmethylsulfonyl fluoride (PMSF) was added at a total concentration of 1 mM to prevent protein degradation. The supernatant was collected after centrifugation at 4 °C and 12,000× *g* for 15 min. The protein concentration was assessed using a BCA Protein Concentration Assay Kit (Beyotime Biotech, Shanghai, China).

The intestinal fluid was extracted from the midgut of third-day fifth-instar silkworms. The activity of AgEGase III in the midgut total protein and intestinal fluid was determined using the 3,5-dinitrosalicylic acid (DNS) method, with procedures adapted from previous studies [29].

### 2.6. Phenotypic Statistics

The developmental times of wild-type silkworm D9L and transgenic silkworm larvae were recorded from the first to the fifth instar. Silkworms were weighed after molting at each instar stage, and the average weight per silkworm was calculated (*n* = 30). At the fifth instar stage, 45 males and 45 females were randomly selected, totaling 90 silkworms for feeding. The daily food intake (mulberry leaves or feed) was recorded, and 24 h later, the larvae were weighed to determine the average weight per silkworm (*n* = 30). Additionally, the remaining food and silkworm excrement weights were measured separately to calculate the average food intake per silkworm, digestion capacity, and digestion rate. Food intake = feeding amount − remaining amount; digestion capacity = food intake − silkworm excrement weight.

The total cocoon weight, cocoon shell weight, and pupa weight of wild-type D9L silkworms and transgenic silkworms were determined, respectively. The average cocoon weight, cocoon shell weight, pupa weight, and cocoon–shell ratio per silkworm (*n* = 30) were calculated. The numbers of eggs laid by wild-type D9L silkworms and transgenic silkworms (*n* = 22) were counted separately. A total of 1000 silkworm eggs were randomly selected and weighed. A total of 10 silkworm eggs from each strain were arranged horizontally and vertically, and their sizes were compared.

### 2.7. Extraction and Detection of Total Protein from Silkworm Eggs

A total of 100 silkworm eggs were randomly selected for total protein extraction. The total protein concentration in the silkworm eggs was measured using the BCA protein concentration assay method. Subsequently, the protein samples were separated by 12% (*w*/*v*) polyacrylamide gel electrophoresis with a sodium dodecyl sulfate-polyacrylamide gel, and the distribution of various proteins in the silkworm eggs was detected using Coomassie brilliant blue staining.

### 2.8. Determination of Cellulose Consumption Rate

The artificial feed and silkworm excrement were dried and weighed on the third day of the fifth instar larvae, and a cellulose (CLL) content detection kit (Solarbio, Beijing, China) was used to determine the cellulose content in the artificial feed and excrement. The specific steps outlined in the manufacturer’s instructions were performed. The consumption rate of cellulose by silkworms was calculated by using the following formula: cellulose consumption = amount of cellulose consumed − the amount of cellulose in silkworm excrement; consumption rate of cellulose (%) = consumption of cellulose/amount of cellulose consumed × 100%.

### 2.9. Determination of Glucose and Reducing Sugar Content

The hemolymph was collected from the silkworms on the first, third, and fifth days of the fifth instar. A glucose content detection kit (Grace Biotechnology, Suzhou, China) was used to measure the hemolymph glucose levels. The DNS method was used to quantify the reduced sugar content in the hemolymph.

### 2.10. Statistical Analysis

The statistical values were reported in the form of mean ± mean standard error (SEM). The mean was compared with the following significance thresholds using Student’s *t*-test: * *p* < 0.05, ** *p* < 0.01, and *** *p* < 0.001. Statistical analyses were performed using GraphPad Prism 8.2 software (GraphPad Software, San Diego, CA, USA) and Microsoft Excel (Microsoft, Redmond, WA, USA). The graphs were drawn using Adobe Photoshop 2022 and Adobe Illustrator 2022.

## 3. Results

### 3.1. Overexpression of AgEGase III in the Transgenic Silkworms

**Figure 1:**
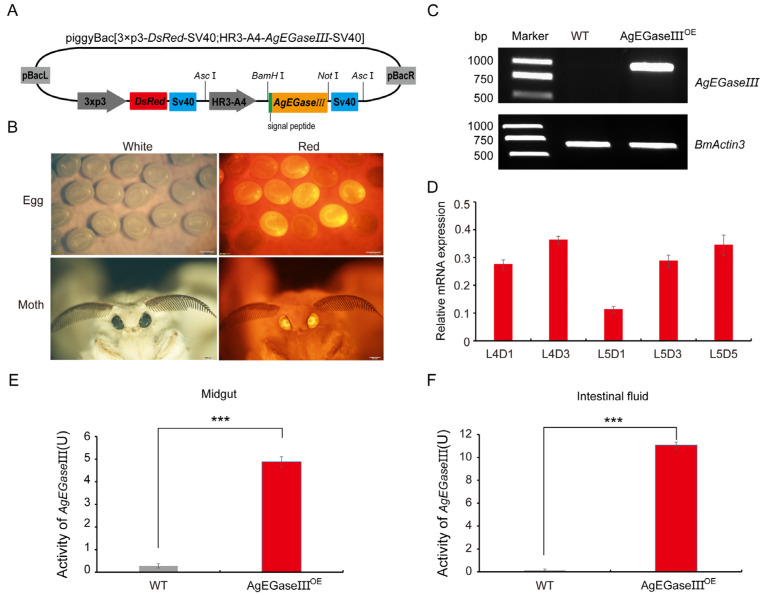
Creation of transgenic AgEGase III-overexpressing silkworms. (**A**) Schematic representation of the transgenic overexpression vector; pBacL and pBacR represent the left and right arms of transposons, respectively. The *AgEGase III* gene is under the control of the HR3-A4 promoter, and a red fluorescent protein is driven by the 3× p3 promoter, serving as a screening marker. (**B**) Transgenic silkworm eggs and moths were observed under white light and red fluorescence. (**C**) Detection of *AgEGase III* gene overexpression in the midgut of transgenic silkworms on the third day of the fifth instar. (**D**) Expression analysis of the *AgEGase III* gene in the transgenic silkworm midgut from the first day of the fourth instar to the fifth day of the fifth instar. (**E**,**F**) Detection of AgEGase III enzyme activity in the midgut (**E**) and intestinal fluid (**F**). WT, wild-type silkworm; AgEGaseIII^OE^, transgenic overexpressed AgEGase III silkworms; ***, *p* < 0.001, *t*-test.

To investigate whether the introduction of the *AgEGase III* gene confers the ability to silkworms to utilize cellulose, a transgenic overexpression vector was constructed using the piggyBac transposon system (Figure 1A). The silkworm eggs and moths displaying red fluorescence in their eyes post the injection of the transgenic overexpression plasmid were confirmed as transgenic silkworms overexpressing AgEGase III, denoted as AgEGase III^OE^ (Figure 1B). The expression characteristics of *AgEGase III* in the midgut of transgenic silkworms were examined, revealing the successful overexpression of the gene in the midgut of transgenic silkworms on the third day of the fifth instar (Figure 1C). Further investigation revealed the sustained expression of *AgEGase III* in the midgut of transgenic silkworms during the late instars (fourth and fifth instars) (Figure 1D).

AgEGase III contains a signal peptide of 22 amino acids at its N-terminus (Figure 1A), enabling its secretion from the cell into the extracellular space [29]. Enzyme activity assays were performed on total proteins in the midgut and intestinal fluid of the transgenic silkworms on the third day of the fifth instar, indicating that compared to the wild type, we detected significant amounts of AgEGaseIII activity in the midgut and intestinal fluid of transgenic silkworms (Figure 1E,F). The enzyme activity characteristics of AgEGase III in transgenic silkworms remained unchanged, with optimal temperature and pH values consistent with previous reports. This further demonstrates that under normal rearing conditions for silkworms (25–28 °C) and an intestinal fluid pH of 8–10, AgEGase III still exhibits high catalytic activity (Figure S1A,B).

Compared to the wild type, the midgut proteins of transgenic silkworms presented significant cellulase activity (Figure 1E and Figure S1C). AgEGase III contains a signal peptide that enables its secretion from the silkworm midgut into the intestinal fluid. The examination of AgEGase III in the intestinal fluid of the transgenic silkworms revealed considerable cellulase activity (Figure 1F and Figure S1D). The enzyme activity characteristics of AgEGase III in transgenic silkworms remained consistent with those reported previously, with negligible changes observed, and their optimal temperature and pH values were essentially the same (Figure S1A,B). This reaffirms that AgEGase III maintains high catalytic activity in silkworms under the standard rearing conditions of 25–28 °C and a midgut pH of 8–10.

### 3.2. Overexpression of AgEGase III Promotes the Growth and Development of Silkworms

**Figure 2:**
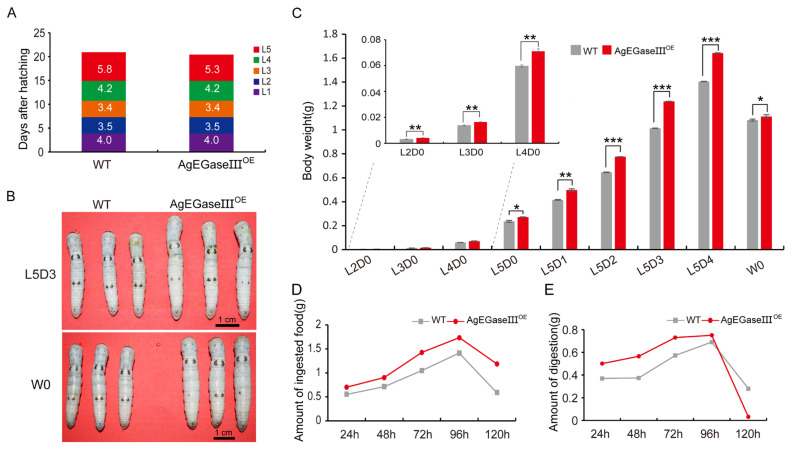
Detection of the developmental duration, larval weight, and feeding of transgenic silkworms. (**A**) Duration of each developmental stage of transgenic silkworms; L1–L5 represent the first to fifth instar stages. (**B**) The phenotype of transgenic silkworms on the third day of the fifth instar (L5D3) and mature silkworm (W0) (scale bar, 1 cm). (**C**) Weight of transgenic silkworm larvae from the second instar (L2D0) to the wandering stage (W0) (*n* = 30). (**D**,**E**) Average food intake (**D**) and digestion amount (**E**) of fifth-instar transgenic silkworms. Digestion amount = food intake − silkworm excrement weight. *, *p* < 0.05; **, *p* < 0.01; ***, *p* < 0.001.

The changes in transgenic silkworms fed mulberry leaves were first examined to assess the impact of AgEGase III overexpression on the development of silkworms. By analyzing the duration of each developmental stage, it was observed that the fifth instar of transgenic silkworms was shortened by 11 h compared to that of wild-type silkworms, whereas the durations of the other instars demonstrated no significant differences (Figure 2A). Phenotypic observations revealed that the body size of the transgenic silkworms was larger than that of the wild-type silkworms on the third day of the fifth instar and wandering stage (Figure 2B). Statistical analysis of the body weights of silkworms at different stages demonstrated that, from the second instar to the pre-pupal stage, the body weight of transgenic silkworms was significantly higher than that of wild-type silkworms (Figure 2C). An investigation into the food intake and digestion of fifth-instar transgenic silkworms fed on mulberry leaves revealed that the food intake of transgenic silkworms was higher than that of wild-type silkworms (Figure 2D). Moreover, the digestion of transgenic silkworms was higher than that of the wild type before 96 h but lower at 120 h (Figure 2E).

**Figure 3:**
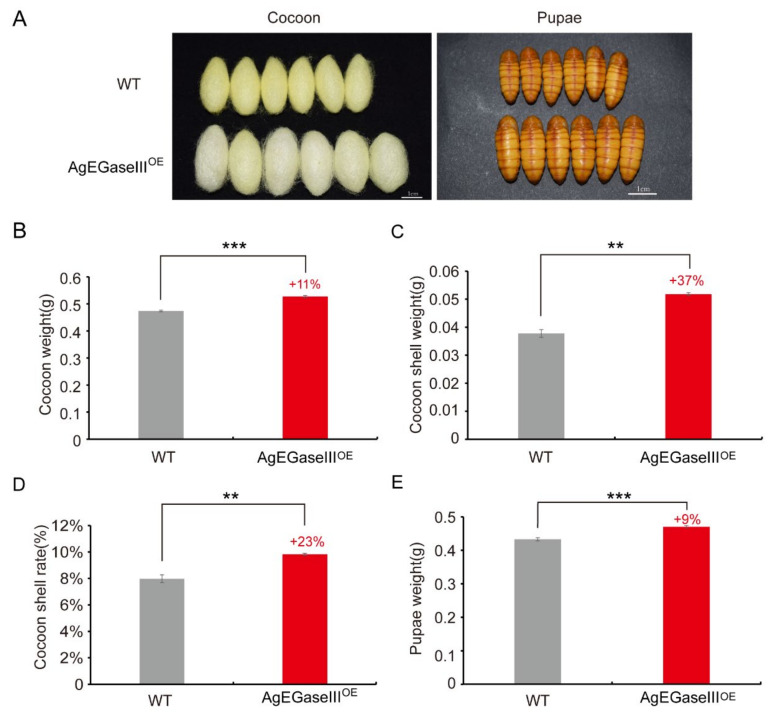
Statistical analysis of the economic characteristics of transgenic silkworm cocoons. (**A**) Cocoons and pupae of transgenic and wild-type silkworms (scale bar, 1 cm). (**B**–**E**) The total cocoon weight (**B**), cocoon shell weight (**C**), cocoon layer rate, and pupal weight (**E**) of transgenic silkworms. WT, wild-type silkworm; AgEGaseIII^OE^, transgenic AgEGase III-overexpressing silkworms. *t*-test, **, *p* < 0.01; ***, *p* < 0.001.

The observation of transgenic silkworm cocoons revealed that their body size and pupae were significantly larger than that of the wild type, with an 11% increase in cocoon weight and a 9% increase in pupa weight (Figure 3A,B,E). Additionally, the cocoon layer weight and rate increased by 37% and 23%, respectively (Figure 3C,D). This suggests that AgEGase III expression in silkworms enhances the economic traits of silkworm cocoons.

**Figure 4:**
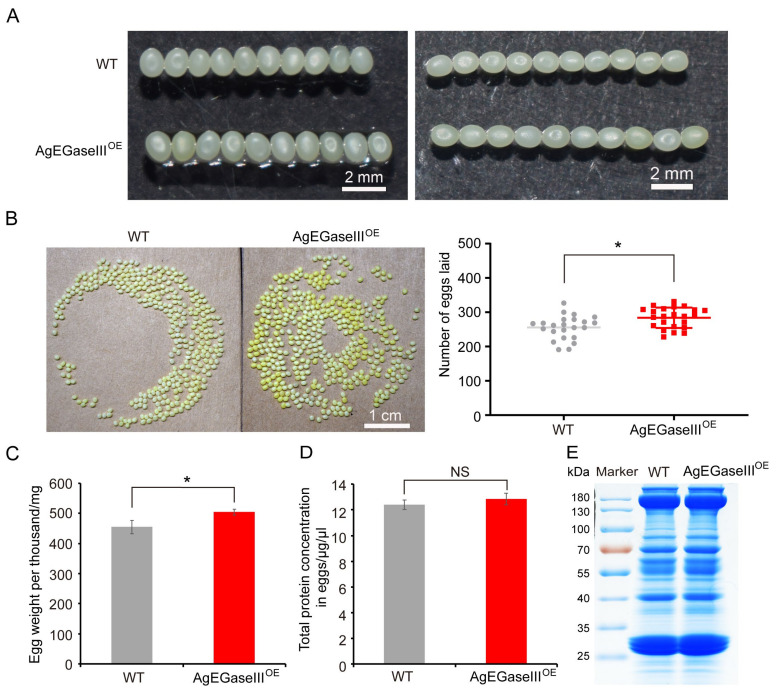
Statistics on the characteristics of transgenic silkworm eggs. (**A**) Vertical and horizontal arrangement of 10 silkworm eggs (scale bar, 2 mm). (**B**) Transgenic silkworm single eggs and egg production statistics (scale bar, 1 cm). (**C**) The thousand-egg weight of transgenic silkworms. (**D**) Total protein concentration of transgenic silkworm eggs. (**E**) Protein profile of transgenic silkworm eggs. WT, wild-type silkworm; AgEGaseIII^OE^, transgenic AgEGase III-overexpressing silkworm. *t*-test, *, *p* < 0.05; NS, not significant.

Ten randomly selected silkworm eggs were arranged both horizontally and vertically, revealing that the egg volume of the transgenic silkworms was larger than that of the wild-type silkworms (Figure 4A). A random survey of 22 single-moth rings in transgenic silkworms demonstrated that the average oviposition number of transgenic silkworms was significantly higher than that of wild-type silkworms (Figure 4B). Further investigation revealed that the weight of 1000 transgenic silkworm eggs was 504.52 mg, whereas that of wild-type silkworms was 454.83 mg. The weight of 1000 transgenic silkworm eggs were significantly increased, by 49.68 mg, compared to that of wild-type silkworm eggs (Figure 4C). The total protein was extracted from 100 transgenic silkworm eggs to analyze the distribution of various proteins. The results demonstrated that the protein content and profiles of transgenic silkworm eggs were similar to those of wild-type silkworm eggs, with no significant differences (Figure 4D,E). This indicated that the introduction of the *AgEGase III* gene did not affect the synthesis and utilization of proteins in silkworm eggs.

The degradation of cellulose by endoglucanases produces cellobiose and glucose. The midgut epithelial cells of silkworms can directly absorb cellobiose and glucose, which are then hydrolyzed into two molecules of glucose by β-glucosidase and released into the hemolymph. Furthermore, the glucose and reducing sugar contents in the hemolymph of transgenic silkworms were measured. The glucose content in the hemolymph of the fifth-instar transgenic silkworms was significantly lower than that of wild-type silkworms (Figure S2A), whereas the content of reducing sugars presented no significant difference compared to wild-type silkworms (Figure S2B).

### 3.3. Overexpression of AgEGase III Enhances the Tolerance of Silkworms to Cellulose

**Figure 5:**
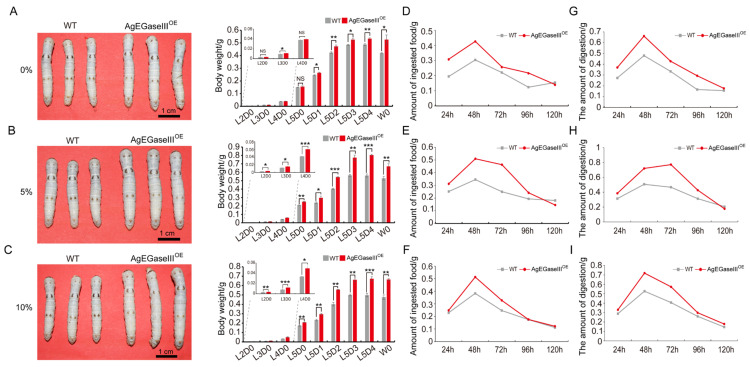
The growth and development of transgenic silkworms reared on artificial feeds with different cellulose concentrations. (**A**–**C**) Observations on the body size and weight gain of transgenic silkworms fed with artificial feeds containing 35% mulberry leaf powder without additional cellulose (**A**), containing 5% cellulose and 30% mulberry leaf powder (**B**), and containing 10% cellulose and 25% mulberry leaf powder (**C**) on the third day of the fifth instar (L5D3). (**D**–**F**) Average food consumption of transgenic silkworms fed with artificial feeds. (**G**–**I**) Average digestion level of transgenic silkworms fed with artificial feeds. WT, wild-type silkworms; AgEGase III^OE^, transgenic AgEGase III-overexpressing silkworms. *t*-test, *, *p* < 0.05; **, *p* < 0.01; ***, *p* < 0.001; NS, not significant.

To further demonstrate the cellulose digestion ability of the transgenic silkworms, they were fed artificial feed supplemented with 5%, 10%, and 15% cellulose. The study findings indicated that, compared to the control group (0% additional cellulose), feeding with artificial feed with 5% and 10% cellulose resulted in a comparable duration of each developmental stage between transgenic and wild-type silkworms (see Figure S3). The comparative analysis of the body size variations in transgenic silkworm larvae reared on the three artificial feeds revealed that all three feed groups resulted in larger body sizes than those of the wild-type silkworms. The statistical analysis of larval weight also demonstrated significantly higher weights for transgenic silkworms reared on artificial feed than for wild-type silkworms. In particular, transgenic silkworms in the 5% and 10% cellulose groups exhibited significantly higher body weights during the second- and third instar stages, with a more pronounced increase in larval body weight as they reached the fifth instar stage (Figure 5A–C). Additionally, the average food consumption (Figure 5D–F) and digestion amount (Figure 5G–I) of fifth instar transgenic silkworms were higher than those of the wild-type silkworms. These findings, which are consistent with those of previous studies on transgenic silkworms fed with mulberry leaves, suggest that the increased body weight of transgenic silkworm larvae may be attributed to an enhanced nutritional intake through increased feeding.

**Figure 6:**
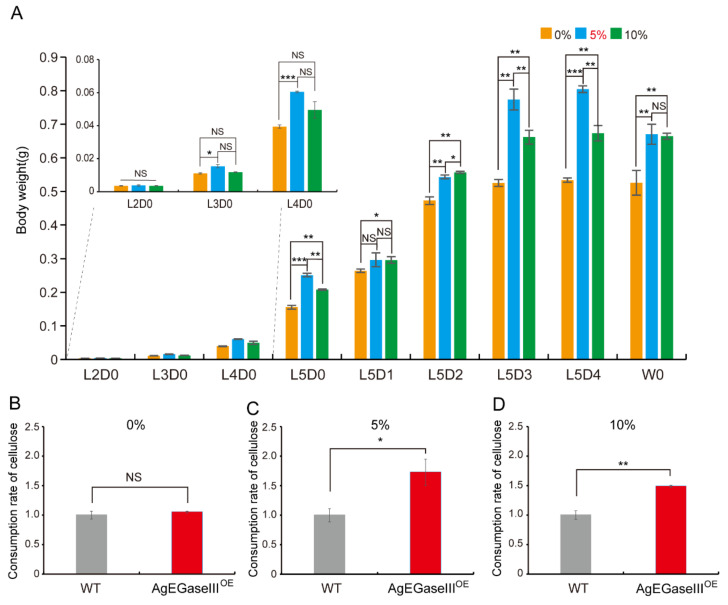
Statistical analysis of transgenic silkworm larval weight and cellulose consumption rate when fed with artificial feed. (**A**) Comparison of transgenic silkworm larval weight when fed with artificial feed containing different cellulose concentrations. (**B**–**D**) Detection of cellulose consumption rate in transgenic silkworms fed with artificial feed containing 0% (**B**), 5% (**C**), and 10% (**D**) cellulose. WT, wild-type silkworms; AgEGaseIII^OE^, transgenic AgEGase III-overexpressing silkworms. *t*-test, *, *p* < 0.05; **, *p* < 0.01; ***, *p* < 0.001; NS, not significant.

The analysis of the weight differences among the three groups of transgenic silkworm larvae revealed that the weights of larvae fed with 5% and 10% cellulose were significantly higher than those fed with 0% cellulose during the fifth instar stage. Furthermore, the larvae fed with 5% cellulose demonstrated significantly greater weight in the third and fourth instar stages (Figure 6A). Statistical analysis of cellulose consumption rates in artificial feed indicated no significant difference between transgenic silkworms fed with 0% cellulose and wild-type silkworms (Figure 6B). However, the cellulose consumption rates of transgenic silkworms fed with 5% and 10% cellulose were markedly higher than those of wild-type silkworms (Figure 6C,D). These results suggest that silkworms overexpressing AgEGase III can effectively break down cellulose in artificial feed.

## 4. Discussion

The mulberry longicorn beetle *A. germari* primarily feeds on mulberry trees, making it a pest that harms them. It possesses three endogenous cellulase enzymes that break down cellulose in the tree trunk into necessary nutrients [27,28,29]. Introducing the *AgEGase III* gene into silkworms resulted in an increased efficiency in digesting mulberry leaf cellulose, leading to improved growth, development, reproductive capacity, and economic traits. Researchers have overexpressed the cellulase gene from the beetle *Batocera horsfieldi* in the silkworm. However, its effects on the growth, development, and economic traits of the silkworm have not been reported [33,34]. This could be attributed to the substrate specificity of cellulase enzymes in *A. germari*, which differs from that of *B. horsfieldi*, as the former feeds on mulberry bark. Therefore, the selection of cellulase genes could benefit from the consideration of insects in future applications for ruminants and other animals whose diet aligns with that of the target animals. For example, exploring cellulase genes from corn borers could be advantageous for transgenic cattle or sheep fed with corn silage.

Research has indicated that substances such as cellulose, hemicellulose, pectin, and lignin in plants act as anti-nutritional factors that can increase the viscosity of the contents in the digestive track of an animal, impeding content flow and subsequently affecting the animal’s feeding behavior, as well as its nutrient digestion and absorption [35]. Transgenic silkworms fed with a mulberry leaf diet exhibited an 11 h reduction in developmental time during the fifth instar stage, along with a significantly larger body size and weight, compared to wild-type silkworms. Moreover, the transgenic silkworms exhibited higher feed intake and digestion levels than the wild type in the fifth instar stage. This enhancement may be attributed to the breakdown of anti-nutritional factors, such as cellulose, in the silkworm gut, leading to the reduced viscosity of the gut contents, as well as improved content flow, increased appetite, and enhanced nutrient digestion and absorption by the silkworms, ultimately resulting in transgenic silkworms consuming more mulberry leaves and promoting their growth and development.

Compared to wild-type silkworms, the total cocoon weight, cocoon-shell weight, cocoon–shell ratio, and pupal weight of transgenic silkworms were significantly increased, by 11%, 37%, 23%, and 9%, respectively. This may be because the transgenic silkworms obtain more nutrients for cocoon formation. The energy reserves in pupae are primarily allocated for reproductive purposes. Generally, the larger the silkworm pupae, the greater the number of silk eggs produced. Since transgenic silkworm pupae are notably larger than wild-type silkworms, the eggs of the transgenic silkworms were larger and heavier. Moreover, the transgenic silkworms had a significantly higher number of eggs per female moth than wild-type silkworms, suggesting that the overexpression of AgEGase III in silkworms can bolster their reproductive capacity.

Mulberry leaves serve as the primary food source for silkworms, and dried mulberry leaf powder is a key component of artificial silkworm feed [36,37]. Mulberry leaves contain a certain amount of cellulose. To explore the cellulose digestion capacity of transgenic silkworms, extra cellulose was supplemented in the base artificial feed, adjusted the cellulose-to mulberry-leaf-powder ratio, and three artificial feeds were created with varying cellulose contents to rear the transgenic silkworms. Silkworms fed with artificial feed containing an additional 5% and 10% cellulose exhibited significantly higher body weights than the 0% cellulose group, with a notably increased cellulose consumption rate compared to the 0% cellulose group. These results further support the ability of transgenic silkworms to digest and absorb cellulose from their diets. Research indicates that there are three sources of enzymes for cellulose digestion in insects: those produced by the insects themselves [29], those produced by microorganisms in the insect gut [38], and a combination of both [39]. Therefore, while the transgenic silkworm still lacks β-exoglucanase, the observed increase in cellulose utilization may be attributed to cellulose-degrading bacteria present in the gut of the transgenic silkworm that provide β-exoglucanase [40]. However, this requires further research in the future. In summary, the overexpression of cellulase in silkworms enhanced their capacity to utilize cellulose from mulberry leaves, thereby benefiting their development and reproductive abilities.

## Data Availability

The original contributions presented in the study are included in the article/Supplementary Materials; further inquiries can be directed to the corresponding authors.

## Supplementary Materials

The following supporting information can be downloaded at: https://www.mdpi.com/article/10.3390/insects15120997/s1, Figure S1: Enzyme activity characterization of AgEGase III in transgenic silkworms. (A,B) Temperature (A) and pH (B) curves of AgEGase III in the hemolymph of transgenic silkworms. (C,D) Enzyme activity detection of AgEGase III in the midgut (C) and intestinal fluid (D) of transgenic silkworms from the first day of the fourth instar to the fifth day of the fifth instar; Figure S2: Determination of reducing sugar content in the hemolymph of transgenic silkworms fed with mulberry leaves. (A) Glucose content (mg/mL) in the hemolymph of transgenic silkworms in the fifth instar stage. (B) Reducing sugar content (mg/mL) in the hemolymph of transgenic silkworms in the fifth instar stage; Figure S3: Duration of each developmental stage of transgenic silkworms fed with artificial feed. (A–C) Duration of each developmental stage of transgenic silkworms fed with artificial feed without additional cellulose (A), with 5% cellulose (B), and with 10% cellulose (C).

## References

[1] Adesogan A.T., Arriola K.G., Jiang Y., Oyebade A., Paula E.M., Pech-Cervantes A.A., Romero J.J., Ferraretto L.F., Vyas D. (2019). Symposium review: Technologies for improving fiber utilization. J. Dairy. Sci..

[2] Deng P., Valentino T., Flythe M.D., Moseley H.N.B., Leachman J.R., Morris A.J., Hennig B. (2021). Untargeted Stable Isotope Probing of the Gut Microbiota Metabolome Using (13)C-Labeled Dietary Fibers. J. Proteome Res..

[3] Dong L., Zhao L., Li B., Gao Y., Yan T., Lund P., Liu Z., Diao Q. (2023). Dietary supplementation with xylooligosaccharides and exogenous enzyme improves milk production, energy utilization efficiency and reduces enteric methane emissions of Jersey cows. J. Anim. Sci. Biotechnol..

[4] Meng Z., Yang C., Leng J., Zhu W., Cheng Y. (2023). Production, purification, characterization and application of two novel endoglucanases from buffalo rumen metagenome. J. Anim. Sci. Biotechnol..

[5] Adesogan A.T., Ma Z.X., Romero J.J., Arriola K.G. (2014). Ruminant Nutrition Symposium: Improving cell wall digestion and animal performance with fibrolytic enzymes. J. Anim. Sci..

[6] Yang J., Refat B., Guevara-Oquendo V.H., Yu P. (2022). Lactational performance, feeding behavior, ruminal fermentation and nutrient digestibility in dairy cows fed whole-plant faba bean silage-based diet with fibrolytic enzyme. Animal.

[7] Yang J., Zhao S., Lin B. (2024). Effect of commercial fibrolytic enzymes application to normal- and slightly lower energy diets on lactational performance, digestibility and plasma nutrients in high-producing dairy cows. Front. Vet. Sci..

[8] Eun J.S., Beauchemin K.A. (2007). Enhancing in vitro degradation of alfalfa hay and corn silage using feed enzymes. J. Dairy. Sci..

[9] Romero J.J., Zarate M.A., Adesogan A.T. (2015). Effect of the dose of exogenous fibrolytic enzyme preparations on preingestive fiber hydrolysis, ruminal fermentation, and in vitro digestibility of bermudagrass haylage. J. Dairy. Sci..

[10] Yang J.C., Guevara-Oquendo V.H., Christensen D., Lardner H.B., Refat B., Rodriguez Espinosa M.E., Yu P. (2023). Utilization of exogenous fibrolytic enzymes in fiber fermentation, degradation, and digestions and characteristics of whole legume faba bean and its plant silage. Crit. Rev. Food Sci. Nutr..

[11] Hammer R.E., Pursel V.G., Rexroad C.E., Wall R.J., Bolt D.J., Ebert K.M., Palmiter R.D., Brinster R.L. (1985). Production of transgenic rabbits, sheep and pigs by microinjection. Nature.

[12] Wu H., Wang Y., Zhang Y., Yang M., Lv J., Liu J., Zhang Y. (2015). TALE nickase-mediated SP110 knockin endows cattle with increased resistance to tuberculosis. Proc. Natl. Acad. Sci. USA.

[13] Monzani P.S., Sangalli J.R., Sampaio R.V., Guemra S., Zanin R., Adona P.R., Berlingieri M.A., Cunha-Filho L.F.C., Mora-Ocampo I.Y., Pirovani C.P. (2024). Human proinsulin production in the milk of transgenic cattle. Biotechnol. J..

[14] Wang Q., Chen X., Xie Z., Liu X., Fu W., Huang K., Xu W., Lin X. (2020). Untargeted Metabonomics of Genetically Modified Cows Expressing Lactoferrin Based on Serum and Milk. J. Agric. Food Chem..

[15] Eriksson S., Jonas E., Rydhmer L., Rocklinsberg H. (2018). Invited review: Breeding and ethical perspectives on genetically modified and genome edited cattle. J. Dairy. Sci..

[16] Meng X., Zhu F., Chen K. (2017). Silkworm: A Promising Model Organism in Life Science. J. Insect Sci..

[17] Chen W., Wang F., Tian C., Wang Y., Xu S., Wang R., Hou K., Zhao P., Yu L., Lu Z. (2018). Transgenic Silkworm-Based Silk Gland Bioreactor for Large Scale Production of Bioactive Human Platelet-Derived Growth Factor (PDGF-BB) in Silk Cocoons. Int. J. Mol. Sci..

[18] Xu S., Wang F., Wang Y., Wang R., Hou K., Tian C., Ji Y., Yang Q., Zhao P., Xia Q. (2019). A silkworm based silk gland bioreactor for high-efficiency production of recombinant human lactoferrin with antibacterial and anti-inflammatory activities. J. Biol. Eng..

[19] Mi J., Zhou Y., Ma S., Zhou X., Xu S., Yang Y., Sun Y., Xia Q., Zhu H., Wang S. (2023). High-strength and ultra-tough whole spider silk fibers spun from transgenic silkworms. Matter.

[20] Batiha G.E., Al-Snafi A.E., Thuwaini M.M., Teibo J.O., Shaheen H.M., Akomolafe A.P., Teibo T.K.A., Al-Kuraishy H.M., Al-Garbeeb A.I., Alexiou A. (2023). *Morus alba*: A comprehensive phytochemical and pharmacological review. Naunyn-Schmiedebergs Arch. Pharmacol..

[21] Percival Zhang Y.H., Himmel M.E., Mielenz J.R. (2006). Outlook for cellulase improvement: Screening and selection strategies. Biotechnol. Adv..

[22] Malik A.D., Furtado I.J. (2022). Isolation of Halomicroarcula pellucida strain GUMF5, an archaeon from the Dead Sea-Israel possessing cellulase. 3 Biotech.

[23] Zhao X., Liu L., Deng Z., Liu S., Yun J., Xiao X., Li H. (2021). Screening, cloning, enzymatic properties of a novel thermostable cellulase enzyme, and its potential application on water hyacinth utilization. Int. Microbiol..

[24] International Silkworm Genome Consortium (2008). The genome of a lepidopteran model insect, the silkworm *Bombyx mori*. Insect Biochem. Mol. Biol..

[25] Watanabe H., Tokuda G. (2010). Cellulolytic systems in insects. Annu. Rev. Entomol..

[26] Byeon G.M., Lee K.S., Gui Z.Z., Kim I., Kang P.D., Lee S.M., Sohn H.D., Jin B.R. (2005). A digestive beta-glucosidase from the silkworm, *Bombyx mori*: cDNA cloning, expression and enzymatic characterization. Comp. Biochem. Physiol. B Biochem. Mol. Biol..

[27] Lee S.J., Kim S.R., Yoon H.J., Kim I., Lee K.S., Je Y.H., Lee S.M., Seo S.J., Dae Sohn H., Jin B.R. (2004). cDNA cloning, expression, and enzymatic activity of a cellulase from the mulberry longicorn beetle, Apriona germari. Comp. Biochem. Physiol. B Biochem. Mol. Biol..

[28] Lee S.J., Lee K.S., Kim S.R., Gui Z.Z., Kim Y.S., Yoon H.J., Kim I., Kang P.D., Sohn H.D., Jin B.R. (2005). A novel cellulase gene from the mulberry longicorn beetle, Apriona germari: Gene structure, expression, and enzymatic activity. Comp. Biochem. Physiol. B Biochem. Mol. Biol..

[29] Wei Y.D., Lee K.S., Gui Z.Z., Yoon H.J., Kim I., Zhang G.Z., Guo X., Sohn H.D., Jin B.R. (2006). Molecular cloning, expression, and enzymatic activity of a novel endogenous cellulase from the mulberry longicorn beetle, Apriona germari. Comp. Biochem. Physiol. B Biochem. Mol. Biol..

[30] Wu J., Li L., Qin D., Chen H., Liu Y., Shen G., Zhao P. (2024). Silkworm Hemolymph and Cocoon Metabolomics Reveals Valine Improves Feed Efficiency of Silkworm Artificial Diet. Insects.

[31] Chen Y., Yao B., Zhu Z., Yi Y., Lin X., Zhang Z., Shen G. (2004). A constitutive super-enhancer: Homologous region 3 of *Bombyx mori* nucleopolyhedrovirus. Biochem. Biophys. Res. Commun..

[32] Shen G., Liu D., Xu H., Wu J., Hou L., Yang C., Xia Q., Lin P. (2023). A Study on the Effect of Energy on the Development of Silkworm Embryos Using an Estrogen-Related Receptor. Int. J. Mol. Sci..

[33] Mei H.Z., Xia D.G., Zhao Q.L., Zhang G.Z., Qiu Z.Y., Qian P., Lu C. (2016). Molecular cloning, expression, purification and characterization of a novel cellulase gene (Bh-EGaseI) in the beetle Batocera horsfieldi. Gene.

[34] Xia D., Wei Y., Zhang G., Zhao Q., Zhang Y., Xiang Z., Lu C. (2013). cDNA cloning, expression, and enzymatic activity of a novel endogenous cellulase from the beetle Batocera horsfieldi. Gene.

[35] Kerr B.J., Shurson G.C. (2013). Strategies to improve fiber utilization in swine. J. Anim. Sci. Biotechnol..

[36] Dong H.L., Zhang S.X., Chen Z.H., Tao H., Li X., Qiu J.F., Cui W.Z., Sima Y.H., Cui W.Z., Xu S.Q. (2018). Differences in gut microbiota between silkworms (*Bombyx mori*) reared on fresh mulberry (*Morus alba* var. *multicaulis*) leaves or an artificial diet. RSC Adv..

[37] Yin X., Zhang Y., Yu D., Li G., Wang X., Wei Y., He C., Liu Y., Li Y., Xu K. (2023). Effects of artificial diet rearing during all instars on silk secretion and gene transcription in *Bombyx mori* (Lepidoptera: Bombycidae). J. Econ. Entomol..

[38] Otagiri M., Lopez C.M., Kitamoto K., Arioka M., Kudo T., Moriya S. (2013). Heterologous expression and characterization of a glycoside hydrolase family 45 endo-beta-1,4-glucanase from a symbiotic protist of the lower termite, Reticulitermes speratus. Appl. Biochem. Biotechnol..

[39] Scharf M.E., Wu-Scharf D., Zhou X., Pittendrigh B.R., Bennett G.W. (2005). Gene expression profiles among immature and adult reproductive castes of the termite *Reticulitermes flavipes*. Insect Mol. Biol..

[40] Li H., Zhang M., Zhang Y., Xu X., Zhao Y., Jiang X., Zhang R., Gui Z. (2023). Characterization of Cellulose-Degrading Bacteria Isolated from Silkworm Excrement and Optimization of Its Cellulase Production. Polymers.

