# Supplementary material for: The Ability to Digest Cellulose Can Significantly Improve the Growth and Development of Silkworms
Source: Insects. 2024 Dec 16;15(12):997. doi: 10.3390/insects15120997 (PMC11678529; doi:10.3390/insects15120997)
Supplement: Supplementary file 1 [file insects-15-00997-s001.zip › insects-3354490-supplementary.pdf]

Supplementary Materials

The Ability to Digest Cellulose Can Significantly Improve the Growth and Development of Silkworms

Jinxin Wu, Yungui Zhang, Han Chen, Qingyou Xia, Ping Zhao, Ying Lin and Guanwang Shen *

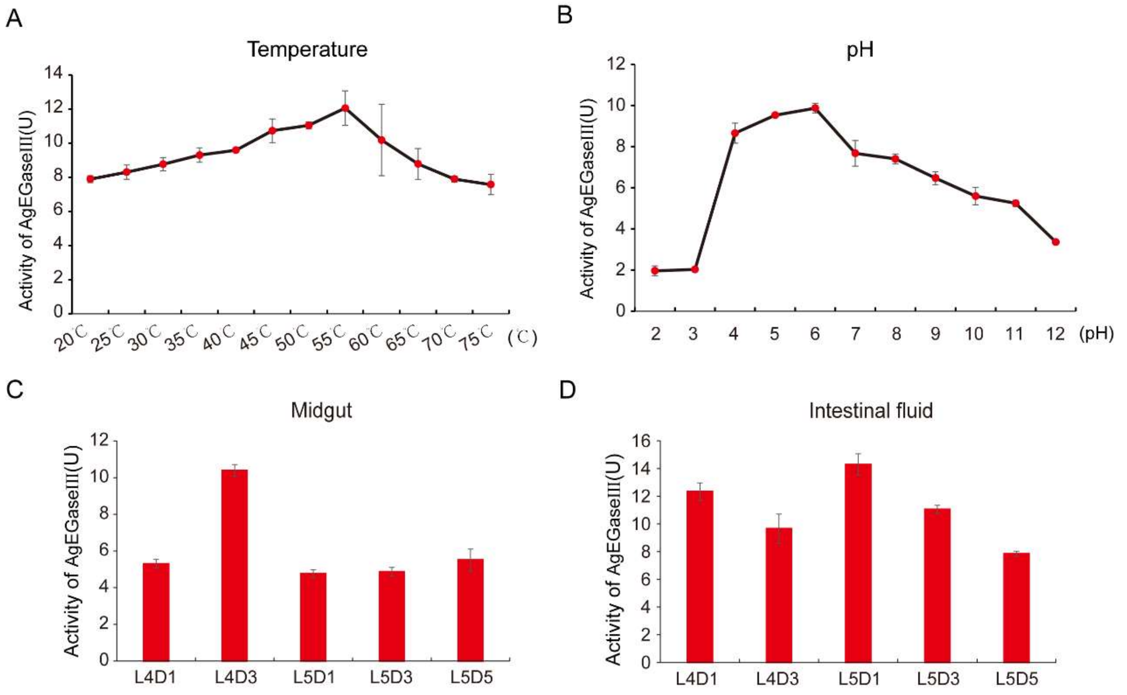

Figure S1. Enzyme activity characterization of AgEGase III in transgenic silkworms.(A–B) Temperature (A) and pH (B) curves of AgEGase III in the hemolymph of transgenic silkworms. (C–D) Enzyme activity detection of AgEGase III in the midgut (C) and intestinal fluid (D) of transgenic silkworms from the first day of the fourth instar to the fifth day of the fifth instar.

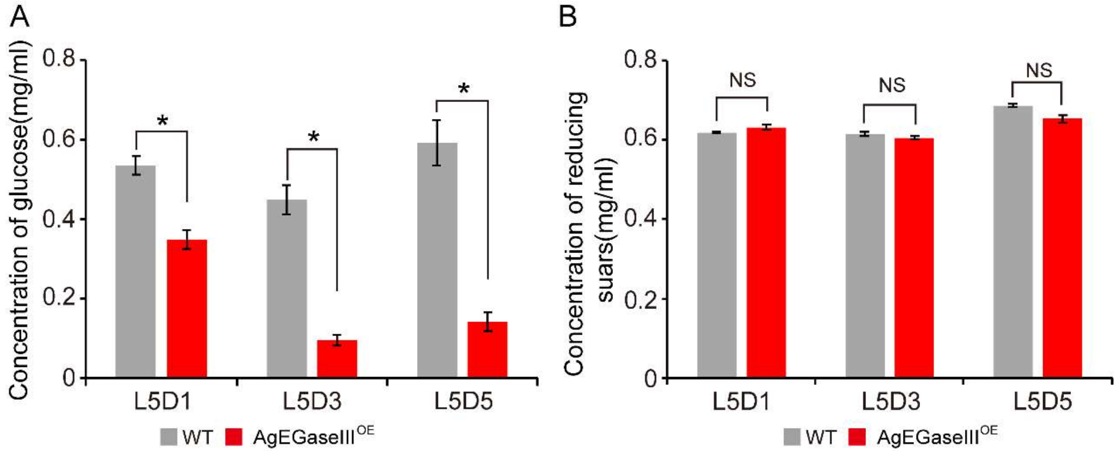

Figure S2. Determination of reducing sugar content in the hemolymph of transgenic silkworms fed with mulberry leaves.(A) Glucose content (mg/ml) in the hemolymph of transgenic silkworms in the fifth instar stage. (B) Reducing sugar content (mg/ml) in the hemolymph of transgenic silkworms in the fifth instar stage. WT, wild-type silkworms; AgEGaseIII^{OE}, transgenic overexpressing AgEGaseIII silkworm. t-test, *, $p<0.05$; NS, not significant.

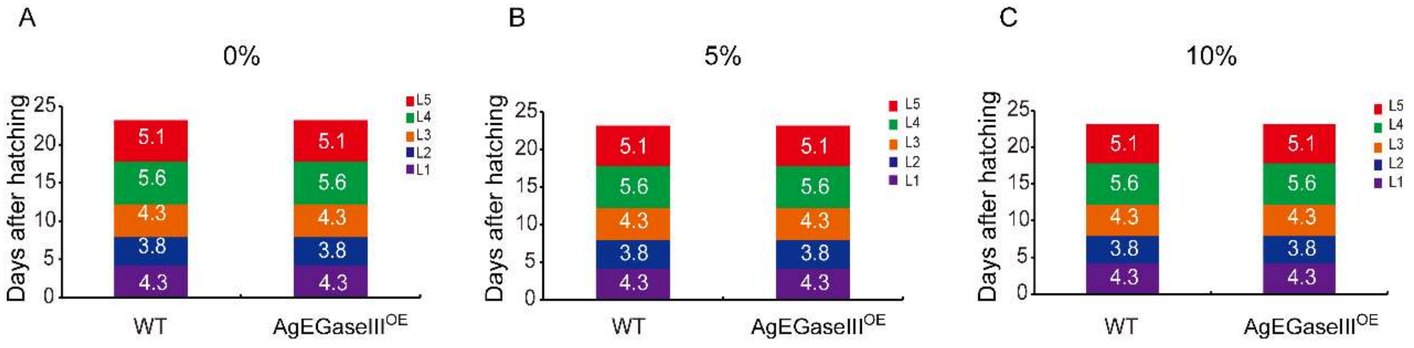

Figure S3. Duration of each developmental stage of transgenic silkworms fed with artificial feed (A–C) Duration of each developmental stage of transgenic silkworms fed with artificial feed without additional cellulose (A), with 5% cellulose (B), and with 10% cellulose (C). WT, wild-type silkworms; AgEGaseIII^{OE}, transgenic overexpressing AgEGaseIII silkworm.
